# The Good and Bad Differentially Encoded within the Subthalamic Nucleus in Rats

**DOI:** 10.1523/ENEURO.0014-15.2015

**Published:** 2015-10-15

**Authors:** Emmanuel Breysse, Yann Pelloux, Christelle Baunez

**Affiliations:** Centre National de la Recherche Scientifique and Aix Marseille Université, Institut de Neurosciences de la Timone Unité Mixte de Recherche 7289, 13385 Marseille, France

**Keywords:** basal ganglia, in vivo electrophysiology, motivation, quinine, reward, sucrose

## Abstract

The subthalamic nucleus (STN) has only recently been added into the reward circuit. It has been shown to encode information regarding rewards (4% sucrose, 32% cocaine). To investigate the encoding of negative value, STN neurons were recorded in rats performing a task using discriminative stimuli predicting various rewards and especially during the replacement of a positive reinforcer (4% sucrose) by an aversive reinforcer (quinine). The results show that STN neurons encode information relative to both positive and aversive reinforcers via specialized subpopulations. The specialization is reset when the context is modified (change from a favorable context (4% vs 32% sucrose) to an unfavorable context (quinine vs 32% sucrose). An excitatory response to the cue light predicting the reward seems to be associated with the preferred situation, suggesting that STN plays a role in encoding the relative value of rewards. STN also seems to play a critical role in the encoding of execution error. Indeed, various subpopulations of neurons responding exclusively at early (i.e., “oops neurons”) or at correct lever release were identified. The oops neurons respond mostly when the preferred reward (32% sucrose) is missed. Furthermore, STN neurons respond to reward omission, suggesting a role in reward prediction error. These properties of STN neurons strengthen its position in the reward circuit as a key cerebral structure through which reward-related processes are mediated. It is particularly important given the fact that STN is the target of surgical treatment for Parkinson’s disease and obsessive compulsive disorders, and has been suggested for the treatment of addiction as well.

## Significance Statement

Subthalamic nucleus (STN) neurons encode information relative to both positive (sucrose) and aversive (quinine) reinforcers via specialized subpopulations of neurons responding to one reinforcer depending on the context (i.e., the reinforcers available). When the context is modified, the specialization of most neurons is reset. An activation of the STN seems to be associated with the most favorable situations. (preferred reward). STN neurons also show reward prediction error-type responses. These properties strengthen its position in the reward circuit as a key cerebral structure through which reward-related processes are mediated. It is particularly important since STN is the target of surgical treatment for Parkinson’s disease (Benabid, 2007) and obsessive compulsive disorders (Mallet et al., 2008) , and has been proposed for the treatment of addiction as well (Pelloux and Baunez, 2013).

## Introduction

Motivation and its neurobiological substrate have been studied largely with a principal focus on the various components of the classic reward system, the dopamine (DA) mesocorticolimbic pathway, the striatum, the nucleus accumbens, ventral pallidum, and prefrontal cortex (PFC). Only in the last decade, has the subthalamic nucleus (STN) been added into the circuit (Baunez et al., 2002). Indeed, former studies in rats have shown that STN lesions or deep brain stimulation (DBS) applied at high frequency decreased the motivation for drugs of abuse such as cocaine, while increasing incentive motivation for food (Baunez et al., 2002, 2005; Rouaud et al., 2010). It was therefore important to further investigate how reward-related information could be encoded by the STN neurons. Incentive motivation has been described as a goal-directed motivation induced by stimuli or cues driving the behavior during the anticipatory period to get the reward (Dickinson and Mackintosh, 1978) or corresponding to the psychological or functional component of the “wanting” for rewards (Berridge, 1996). Reward-related information is thus not expected to be encoded in the STN only at the moment of reward delivery, but also at the presentation of the cue. Indeed, in cue-directed instrumental conditioning or associative learning, the association of a positive or negative unconditioned stimulus (sucrose or quinine) with a conditioned stimulus (e.g., lights) attaches positive or negative value to that conditioned stimulus (Konorski, 1948; Skinner, 1966a,b). Thus, associating a cue light with quinine will confer negative value to the cue light predicting quinine. Indeed, it has been shown that STN neuronal activity could be modulated by different positive reinforcers (various concentrations of sucrose or cocaine, and fruit juice) at delivery and by their associated predicting cues whatever the nature of the rewards (Darbaky et al., 2005; Espinosa-Parrilla et al., 2013; Lardeux et al., 2009, 2013). In order to further assess the role of STN in motivational processes, and in encoding aversive stimuli, here the responses of STN neurons have been studied during the replacement of a positive reinforcer (sucrose) by an aversive reinforcer (quinine) during the same session. This also allowed further analyses of STN neuronal adaptation to the change from a favorable context with only positive reinforcers (4% vs 32% sucrose) to an unfavorable context (quinine vs 32% sucrose).

In addition to its involvement in reward processing, past studies have shown that STN lesion, in the rat, increases impulsivity (Baunez et al., 1995; Baunez and Robbins, 1997; Phillips and Brown, 1999), suggesting that the STN also plays a role in the control of inhibition (for review, see Eagle and Baunez, 2010). However, in order to select the action to inhibit, the brain must have a system to differentiate correct actions from incorrect ones. Recent work has shown that STN neurons could encode error of execution in rats (i.e. “oops neurons”), exhibiting a different response during correct and incorrect responses when working for positive reinforcers (Lardeux et al., 2009, 2013). Moreover, the STN has also revealed its ability to adapt its neuronal responses in case of challenges, when the reward expected was modified unexpectedly, suggesting a role for STN in reward prediction error (RPE; Lardeux et al., 2009, 2013). Since STN receives afferences from dopaminergic neurons of substantia nigra/VTA, which are known to encode RPE (Schultz, 1998; Brischoux et al., 2009; Matsumoto and Hikosaka, 2009), it might well be possible that these afferences are responsible for the STN adaptation to new rewards. To further assess the STN neuronal responses to a negative reward prediction error, reward omission was tested. STN neurons were recorded in freely moving rats performing a customized task using discriminative stimuli predicting various rewards, but the announced reward was omitted in some trials.

## Material and Methods

### Animals

For both the electrophysiology and the taste reactivity experiments, Lister Hooded male rats (*n* = 10 and *n* = 8, respectively; Janvier Labs) were used and kept in pairs in Plexiglas cages (42 × 26.5 × 18.5 cm) in the animal facility with the lights on from 8:00 A.M. to 8:00 P.M. They were handled upon arrival to habituate them to contact with the experimenter. In all training and recording sessions of the electrophysiological experiment, the rats had access to water without restriction, while their food was limited (12-15 g/d/rat) to keep them motivated. Each day, rats were weighed. At the moment of the surgery, their weights ranged between 380 and 420 g. Once implanted, the rats were placed individually in their cages to prevent them from damaging their headcap. For the taste reactivity experiment, the rats had access to water and food without restriction, and were kept in pairs before the implantation of the oral fistula. After surgery, they were singly housed to prevent other rats damaging their implants. All procedures, animal handling, and surgery were conducted in accordance with and were approved by the French Agriculture and Forestry Ministry (Decree 87-849).

### Surgery

#### Electrophysiology

After the rats were trained in the behavioral task and had good and stable behavioral performances, they were anesthetized with a mixture of ketamine (Imalgène 1000; 100 mg/kg, i.m.) and medetomidine (Domitor; 250 mg/kg, i.m.). After the surgery, they received a dose of atipemazole (Antisédan; 75 mg/kg, i.m.) to cancel the effects of medetomidine. Two bilateral, drivable multiwire electrode bundles of four tetrodes were stereotaxically positioned just above the STN. Coordinates were established in millimeters at the electrode tip, which were chosen to target the dorsal limit of the STN (anteroposterior, −3.7, lateral, ±2.4 from the bregma; dorsoventral, −8.15 relative to the surface of the skull above the target point (Paxinos and Watson, 2005). The drivable electrode assemblies lowered vertically at coordinates previously calculated, and their supports were anchored to the skull with four stainless steel screws and dental cement. After the surgery, animals had 1 week of recovery.

#### Taste reactivity

The rats received oral catheter implantation according to McCutcheon et al. (2012), allowing the injection of different liquids directly inside their mouth. The rats were anesthetized in the same way as previously described. The cannula, consisting of PE-100 tubing with one tip flared and secured with a Teflon washer, and the other tip attached to a needle. The needle was then inserted into the internal side of the cheek at the level of the first maxillary molar and run with the tubing under the skin along the zygomatic arcade and exteriorized out the incision at the top of the head. The top end of the cannula was then held in place with a second Teflon washer.

### Apparatus

#### Electrophysiology

Training and recording sessions took place in two custom-built Plexiglas operant boxes (Med Associates). A porcelain retractable lever and two cue lights, one on either side of the lever, were located along one wall. The box was also equipped with a feeder, located opposite the lever, comprising two magazines where the sucrose (32% or 4%) and quinine (0.02 m; Setlow et al. 2003) solution (0.1 ml/trial) were delivered. A Tygon tubing catheter connected to a 10 ml syringe mounted on a fixed displacement pump (Med Associates) and connected to each cup allowed reward delivery. One tone generator (3.5 kHz) provided a nonlateralized auditory stimulus. Ambient light (house light) was on at the beginning of the session and was turned off at the end of the session or during errors. An interface (MedPC, Med Associates), and a computer controlled the session and collected the electrophysiological signals, which were transmitted to a preamplifier that was located on the headstage, then to amplifiers (Neuralynx) and to data-acquisition hardware (Datawave Technologies).

#### Taste reactivity

Habituation and testing took place in an elevated Plexiglas cylinder (96 cm above the ground, 20 cm in diameter, and 30 cm in height) elevated 0.5 cm from a Plexiglas floor under which a camera was mounted and connected to a DVD recorder. A 10 ml syringe mounted on a pump (Razel) could be connected to the fistula via Tygon tubing.

### Behavioral procedure

#### Electrophysiology

The animals were conditioned to perform a cued simple reaction time (RT) task with the cue providing information regarding the reward to be obtained. Two designs were used: the first to test whether the STN could encode the expectation of an aversive reinforcing agent (quinine; challenge 1), and the second to test whether the STN encodes reward omission [when a reward (4% and 32% sucrose) is expected but not delivered; challenge 2].

#### Standard condition

**Figure 1.**
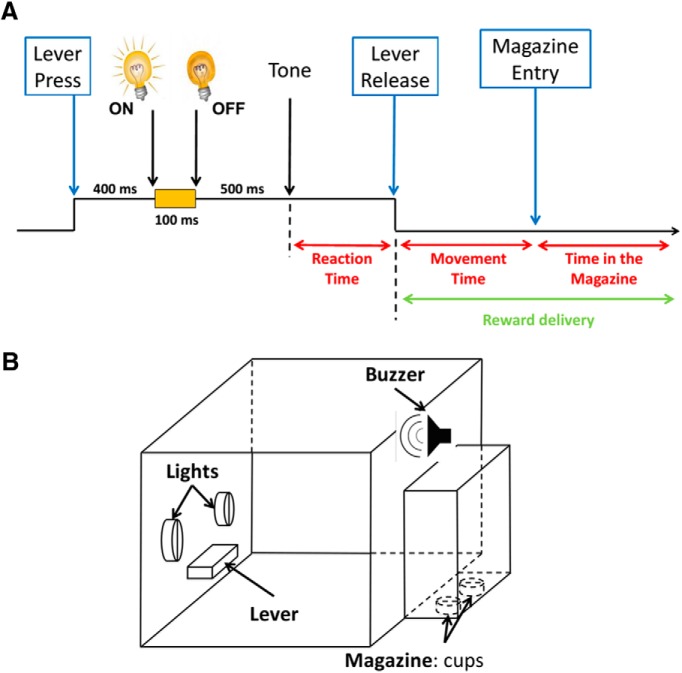
Behavioral task. ***A***, This diagram illustrates the time elapsing during one trial (black arrow). The rats had to press the lever down for 1 s. During this 1 s period, after 400 ms had elapsed, one cue light was switched ON (either left or right) for 100 ms, providing information regarding the future reward (Left light, 4% sucrose or quinine; Right light, 32% sucrose). The rats had to maintain their paw on the lever until the end of the 1 s period (i.e., an extra 500 ms) that was signaled by a tone. The rewards were then delivered after the rats had withdrawn the lever. Reaction Time is the time between the trigger tone and the lever release; Movement Time is the time between the lever release and the detection of the nose of the rat in the magazine; and the time spent in the magazine after reward delivery (i.e., from detection of the nose of the rat until withdrawal) was measured as the consumption time. ***B***, Operant box in which animals were trained and recorded. The box is equipped with two lights, one lever, one buzzer, and one magazine with two cups.

At the beginning of the session, the house light was turned on and the lever extended. Rats were trained to press the lever for 1 s. Forcing the animal to press the lever and hold it down until the tone occurred involved the inclusion of the motor-related activity of STN in the baseline, thereby allowing the selective neural activity related to other events (light and tone) to be revealed. During this period and 400 ms after the start of the lever press, one of the two cue lights was randomly illuminated for 100 ms. A trigger tone was delivered at the end of the 1 s interval, indicating that the rat could release the lever and the cued reward was delivered (Fig. 1). Each cue light (either right or left of the lever) was associated with a specific reward as long as the response was correct (i.e., lever released after the tone). Half of the rats were trained with the following rule: left light indicated that 4% sucrose was the reward; and the right light indicated that the reward was 32% sucrose. The other rats were trained with the opposite rule (i.e., left light, sucrose 32%; right light, sucrose 4%). Immediately after the rat released it, the lever was retracted and the pump was activated. The detection of the head entry of the animal in the magazine after a correct lever release began a 5 s intertrial interval. Anticipatory lever releases (release before the trigger tone) were not rewarded, and led to the retraction of the lever and to the extinction of the house light for 5 s. Each session ended after 120 trials (60 trials with each reward randomly distributed) or if 30 min had elapsed. All behavioral responses were time stamped through an electrophysiological recording system for later analysis of event-related neuronal activity.

##### Challenge 1: 32% sucrose versus quinine

Each recording session began with the standard condition. Then 4% sucrose was replaced by quinine after 15 min had elapsed or 60 trials were performed, leading to the possibility for the animal to get either 32% sucrose or quinine (0.01 m): this was the challenge condition. The animals (*n* = 10) were subjected to this challenge for 20–30 sessions (except for two rats, which lost their electrodes and have been recorded for only 12 and 13 sessions under the challenge 1 condition). Importantly, as few attempts to replace 32% sucrose by quinine led the animals to completely stop working on the task, the challenge condition presented here consisted only of the replacement of the 4% sucrose solution.

##### Challenge 2: reward omission

Rats had to perform the same behavioral task working for 32% and 4% sucrose, but in 20% of the trials (24 trials; i.e., 12 for each reward), the reward was not delivered.

#### Taste reactivity

Once connected, rats were placed into the cylinder for 2 min. The pump was then activated for 30 s, delivering a 1 ml passive infusion of water for habituation. Habituation was repeated the following day. On day 3, three 30 s infusions of increasing concentrations of sucrose (4%, 10%, and 32% w/v) were then performed, as previously described, and were interspaced with 2 min washout periods. On day 4, two 30 s infusions of increasing concentrations of quinine (0.01 and 0.02 m) were tested following the same procedure.

### Manufacture of electrodes

Each animal was implanted with four tetrodes (4 × 4 electrodes). Each tetrode was made by twisting four threads of nickel-chrome that were 25 μm in diameter. Once the four threads were twisted, one tip was stuck between two mill-max pins, allowing electrical connection (SuperShield). They were then inserted into the plastic of a mill-max, and the four tetrodes were inserted into two stainless steel cannulae of 0.5 mm in diameter (26 gauge) and 28 mm long, which served as the ground. The assembly was then placed on a self-made drivable support and sealed with resin (Orthoresin) to protect and secure the assembly. The drivable support was inspired by another drivable system developed for behaving rats (du Hoffmann et al., 2011).

### Recording

After 1 week of recovery following surgery, the rats were returned to the training schedule until they reached their level of preoperative performance. Then they were recorded each day during the behavioral sessions. The recording of neuronal activity began every day before the start of the behavioral session, so that the threshold on the various channels could be determined. All waveforms exceeding an amplitude threshold (1.5 times above the background noise level) were recorded. Since recordings were performed with drivable tetrodes, each day the screws moving the tetrodes were turned so that they were moved ∼32 µm lower (or higher). The aim was to reduce the probability of recording the same neuron and to explore more STN neurons. Among the multiple recording sessions, the tetrodes were first moved down, since they were placed at the upper boundary of the STN calculated from the implantation site, and then were moved up once the lower boundary of the STN, calculated based on the atlas, was reached.

### Data analysis

#### Behavioral analyses

Different task performance, RT (i.e., the time taken for the animal to release the lever after the tone), and movement time (MT; i.e., the time taken for the animal to enter the magazine after releasing the lever) were determined. These two measures provided an indication of whether the animals understood the incentive value of the various cue lights. Optimal discrimination between the two lights and their associated reward should lead to shorter MTs and RTs for the preferred reward (Hauber et al., 2000). The time spent in the magazine, or consumption time (CT), provided further evidence of reward-relative preference. The preferred reward was mostly consumed entirely (with therefore more time spent in the magazine than for the less preferred reward), while the less preferred reward was not always consumed or at least was not totally consumed. The RT, MT, and CT were analyzed independently in the standard condition and during the challenges with quinine. For each measure, the equality of variance was verified with a Fisher’s test. When this was not fullfilled (*F* test < 0.05), a logarithmic transformation was performed prior to comparison by ANOVA and *post hoc t* test using StatView (SAS Institute). In order to analyze the interaction between both conditions, ANOVA with two factors (conditions and reinforcers as within-subject factors) was performed.

For the taste reactivity, hedonic responses (rhythmic tongue protrusions) and aversive responses (gapes) were counted manually over the 30 s infusions from video recording across the different concentrations of either sucrose or quinine (4%, 10%, and 32% sucrose; 0.01 and 0.02 m quinine). The effect of concentration was tested with a one-way repeated-measures ANOVA with the concentration as the within-subject factor. For each concentration, a Wilcoxon signed rank test was used to compare the number of gapes to zero. A *p* value <0.05 was taken to be a significant difference.

#### Electrophysiological analyses

Spike sorting was performed off-line using SciWorks clustering software (Datawave Technologies). Analyses were based on binned perievent firing rates (50 ms bins) obtained for each session. For each event, a perievent time histogram (PETH), centered on that event, was made with NeuroExplorer. For all rewards, the neuronal responses to lever press, cue light, lever release, and magazine entry were analyzed separately. The responses to the cue light and at the lever release were analyzed separately for correct and incorrect trials (i.e., when lever release occurred before the trigger tone). In many cases, more than one waveform shape could be isolated on a single wire. When these waveforms could not be easily separated, they were discarded from the analysis. Autocorrelograms were then constructed for each unit using NeuroExplorer (Nex Technologies), and units without 2 ms refractory periods were either rejected or resorted. Although a sample of several hundred units was recorded, it is likely that some signals were recorded more than once from the same neuron over the course of the experiment across the different sessions. In this case, to avoid analyzing the same neuron twice in consecutive sessions, waveforms of neurons recorded on the same electrode were compared according to the analysis b Grossman et al. (2008). Briefly, waveforms recorded from the same electrode were considered as originating from different neurons if multivariate ANOVAs making comparisons across two consecutive sessions showed significant a difference with *p* < 0.001. Otherwise, they were considered as originating from the same neuron, and the second recording of this neuron was discarded from the analysis. Finally, to minimize the contamination of signals by activity related to a previous event, the neural response to each event was analyzed across the 500 ms starting at this event and was compared with the activity over the 400 ms preceding the event itself. The 400 ms baseline interval was chosen to maximize sampling but also to prevent event-related activity to collide by using the shortest period between two consecutive events (the lever press and the illumination of the cue light). Analyses were performed according to the analysis of Teagarden and Rebec (2007). Briefly, the mean firing rate for each perievent bin was expressed as a *z*-score (zi) based on the following formula:

$zi=\frac{Fqi-mean(baseline)}{SEM(baseline)}$,with Fqi as the mean firing rate (in hertz) of the bin (*i*) and the basal period (baseline) preceding each event interval corresponding to 400 ms before the event [−400:0 ms]. Three or more consecutive bins (≥150 ms) with *z*-scores ≥1.64 SD (95% confidence interval) were considered to be significant activation or inhibition. Finally, for each event, neurons were classified in different categories (“similar” and “specific”). The firing rate of neurons that responded for an event was compared for each reward with a *t* test on the normalized data. Thus, neurons were similar if they responded to one event in a similar manner for both rewards (*t* test, *p* > 0.05). Neurons were specific if they responded to one event for both rewards with a significantly higher response to one reward than the other or if they responded exclusively to one reward. For example, one neuron that responded more after the cue light announcing the 32% sucrose than that announcing the 4% sucrose was classified as “specific 32% sucrose” at the cue light. The proportions of neuronal subpopulations (e.g., “selective 32%” vs “selective 4%”) expressed in percentages were compared using a χ^2^ test. The average of the *z*-score of the population PETH have been illustrated by separating the specificity of neurons for either each reward or correct versus incorrect trials, based on the previously defined criteria (three bins with *z*-scores ≥1.64 SD). The neurons also have been analyzed depending on their type of response (activation or inhibition) to the events (cue light and lever release), and the *z*-scores have been calculated. The percentage of variation for activated and inhibited neuronal populations has then been calculated comparing the mean firing rate before [−400:0 ms] and after [0:500 ms] each event.

### Histology

At the end of the experiment, all rats were killed; and the brains were removed, frozen, and cut into coronal sections with a cryostat. Frontal 30-µm-thick sections of the STN were stained with cresyl violet for assessment of the electrode placement. The lower recording site was identified, and reconstruction of the various recording sites was estimated according to the distance covered by the number of screw rotations.

## Results

### Histology

**Figure 2.**
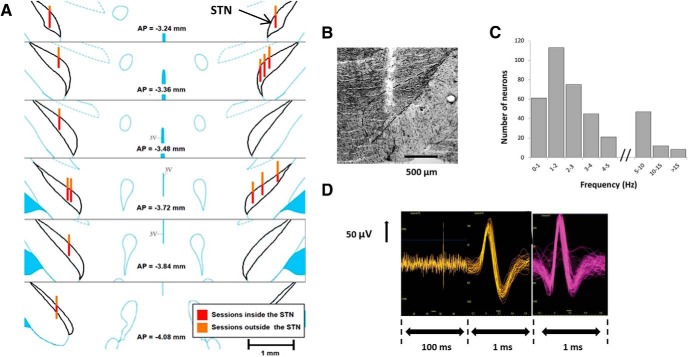
Histological, neuronal, and waveform characterization. ***A***, Estimation of the placement of the 14 electrodes inside the STN. Sessions associated with the red track (inside the STN) were kept in the analysis, while those associated with the orange track (outside of the STN) were discarded. AP = −3.24 to −4.08: anteroposterior levels taken from the atlas of Paxinos and Watson (2005). Scale bar, 1 mm. ***B***, Cresyl violet staining showing an electrode track inside the STN (delimited by the black dashed line). Scale bar, 500 µm. ***C***, Distribution of the 382 STN neurons according to their mean firing rate (Hz). ***D***, Example of different waveforms of some representative neurons recorded in the STN showing one spike (left), biphasic waveforms (middle), and triphasic waveforms (right).

Fourteen of the 20 electrode bundles implanted in the 10 rats had their tip in the STN. Data from these 14 electrode bundles were kept for the analysis, providing that the recorded session was within the boundaries of the STN. In most cases, the electrodes were in the anterior part of the STN (Fig. 2*A*). The track was estimated for each drivable bundle, in line with the number of movements of the screws made in each direction (down and then up). The neurons recorded outside the boundaries of STN were discarded (*n* = 67; Fig. 2*A*, estimated in yellow tracks).

### Behavioral results

#### Taste reactivity

**Figure 3.**
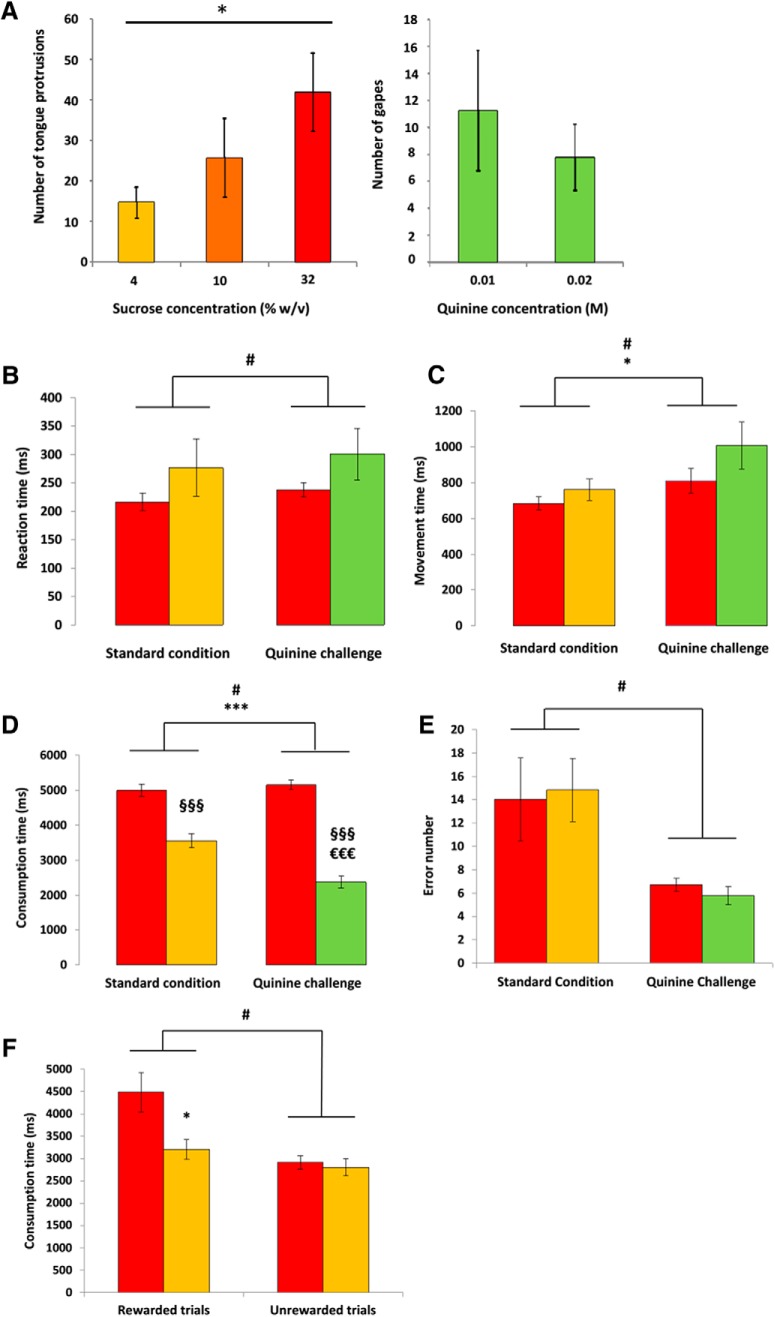
Behavioral results. ***A***, Taste reactivity measure. Positive responses (tongue protrusions) induced by various concentrations of sucrose [left; 32% sucrose (red), 10% sucrose (dark orange), 4% sucrose (orange)] and negative responses (gapes) induced by two different concentrations of quinine (right; green). *Significant concentration effect (*p* < 0.05). ***B***, Mean Reaction Time (time to release the lever after the tone onset in milliseconds ±SEM) for 32% sucrose (red bars), 4% sucrose (orange bars), and quinine (green bars) in the standard condition and during the quinine challenge. #Significant challenge effect (*p* < 0.05). ***C***, Mean Movement Time (time to reach the magazine after the lever release in milliseconds ±SEM) for 32% sucrose (red bars), 4% sucrose (orange bars), and quinine (green bars) in both standard and quinine challenge conditions. *Significant reward effect; #Significant challenge effect (*p* < 0.05). ***D***, Mean Consumption Time (CT) (time spent in the magazine after reward delivery in milliseconds ±SEM) for 32% sucrose (red bars), 4% sucrose (orange bars), and quinine (green bars) in both standard and quinine challenge conditions. #Significant challenge effect (*p* < 0.05); ***significant reward effect (*p* < 0.001); §§§significantly different from 32% sucrose (*p* < 0.001); €€€significantly different from 4% sucrose (*p* < 0.001). ***E***, Mean number of errors (premature lever release) after the cue light onset for 32% sucrose (red bars), 4% sucrose (orange bars), and quinine (green bars) in both standard and quinine challenge conditions. #Significant challenge effect (*p* < 0.05). ***F***, Mean CT (in milliseconds ±SEM) during challenge 2 for 32% sucrose (red bars), 4% sucrose (orange bars), when the reward was delivered (rewarded trials) vs when for 20% of the successful trials the reward was omitted (unrewarded trials). *Significant reward effect (*p* < 0.05); #significant challenge effect (*p* < 0.05).

While hedonic responses (rhythmic tongue protrusions) dose-dependently increased with the various concentrations of sucrose (concentration effect: *F*_(8,16)_ = 7.08, *p* = 0.0063), the quinine induced aversive-type responses (Wilcoxon test, *p* < 0.05 for each concentration of quinine) independent of the dose tested [concentration effect: *F*_(8,8)_ = 1.4, *p* = 0.4433 (NS)] such as “gapes,” “rubbings,” and “wetdog shakes” within the 30 s following its administration (Fig. 3*A*). Moreover, the quinine induced no tongue protrusions compared with the 4%, 10%, and 32% sucrose (mean, 15%, 26%, and 42% vs 0, respectively; *t* test: *t* = 3.901, 2.595, and 4.355; *p* < 0.05), and the sucrose solution induced no gapes whatever the sucrose concentration compared to quinine solution (mean = 0 vs 8 ± 2.61; *t* test: *t* = −3.162, *p* < 0.05; data not shown). These results strongly demonstrate that rats found the solution of sucrose pleasurable at the concentrations used (4%, 10%, and 32%) but did not like the quinine concentrations (0.01 and 0.02 m). This suggests that quinine at the concentration used for the electrophysiology experiment can be considered to be aversive for rats.

#### Reaction time task

##### Challenge 1: 32% sucrose versus quinine

Under standard conditions (sucrose 4% vs 32%), the rats showed a trend to take longer to release the lever after the trigger tone for the sucrose 4% solution than for the 32% solution (mean ± SEM: 276.9 ± 47.7 and 216.4 ± 14.5 ms, respectively). When sucrose 4% was replaced by quinine, the RT for quinine increased further, although the difference between quinine and sucrose 32% did not reach significance [mean ± SEM: 301.3 ± 42.8 and 237.9 ± 12 ms, respectively; reward effect: *F*_(1,8)_ = 2.928; *p* = 0.1254 (NS)], possibly because that for sucrose 32% increased as well (challenge effect: *F*_(1,8)_ = 8.154; *p* = 0.0213; Fig. 3*B*). In line with the RT observations, in the standard condition, the rats were slower to reach the magazine (MT measures) for 4% sucrose than for 32% (Fig. 3*C*; mean ± SEM: 759 ± 57.1 and 682.8 ± 32.7 ms, respectively). As expected, when quinine replaced sucrose 4%, the rats were also slower to reach the magazine to collect quinine than to collect 32% sucrose (Fig. 3*C*; mean ± SEM: 1005.2 ± 124 and 810.9 ± 65 ms, respectively). In each condition, the rats were slower for the less preferred solution (4% sucrose or quinine) than for the 32% sucrose, the supposedly preferred solution (reward effect: *F*_(1,8)_ = 7.635; *p* = 0.0246), suggesting that the rats have properly associated the different lights with each reinforcer. As for RTs, the quinine induced a general increase in MTs (challenge effect: *F*_(1,8)_ = 9.149; *p* = 0.0164). The measure of the time spent in the magazine (consumption time) revealed that, in the standard condition, the rats spent less time licking the 4% sucrose than the 32% sucrose (mean ± SEM: 3550 ± 188 and 4990 ± 165 ms, respectively; Fig. 3*D*, left). So did they for quinine when it replaced the 4% sucrose (mean ± SEM: 2369 ± 471 and 5145 ± 131 ms, respectively; Fig. 3*D*, right), and also less than the 4% sucrose in standard condition (interaction reward × challenge effect: *F*_(1,8)_ = 181.4; *p* < 0.0001; Fig. 3*D*). In fact, the animals did not consume the quinine solution, suggesting that sucrose 32% is always the preferred solution, whatever the condition, as previously shown (Lardeux et al., 2009, 2013), but also that quinine is worse than 4% sucrose (challenge effect: *F*_(1,8)_ = 12.974; *p* = 0.007; and interaction reward × challenge effect: *F*_(1,8)_ = 181.4; *p* < 0.0001, respectively). Surprisingly, the rats made fewer errors during the quinine challenge than during the standard condition (challenge effect: *F*_(1,8)_ = 9.018; *p* = 0.017; Fig. 3*E*).

##### Challenge 2: reward omission

Because the omission trials were unsignaled and randomly assigned, it was not surprising to observe no difference between standard and omission trials in terms of RT, MT, and errors (data not shown). In terms of CT, as shown in the standard condition (Fig. 3*D*, left), the rats spent more time in the magazine for the 32% sucrose than for the 4% sucrose when the reward was delivered (mean ± SEM: 4095 ± 308 and 3259 ± 142 ms, respectively; Fig. 3*F*, left). However, the significant interaction between challenge and reward (interaction effect: *F*_(1,8)_ = 5.757; *p* = 0.0432) reflected that when the reward was omitted, the rats spent the same low amount of time in the magazine, whichever reward was omitted (mean ± SEM: 2989 ± 194 and 2803 ± 221 ms, respectively; challenge effect: *F*_(1,8)_ = 6.423; *p* = 0.035; Fig. 3*F*, right).

### Electrophysiological results

A total of 382 STN neurons were recorded. The mean frequency of these neurons was 3.32 ± 0.80 Hz, close to the mean STN frequency found by Teagarden and Rebec (2007). The majority of the discharging neurons had an average frequency of between 1 and 6 Hz (Fig. 2*C*), which is in line with what was described previously (Lardeux et al., 2009, 2013). The duration of mean action potentials (APs) was ∼0.8 ms, and the amplitude of AP varied from 50 to 150 µV, and with a biphasic waveform (Fig. 2*D*, left). The waveforms of the APs were biphasic with a first positive activity followed by a negative activity (Fig. 2*D*). Some triphasic waveforms, according to Teagarden and Rebec (2007), were also found. Moreover, sometimes APs with initial-segment-soma-dendritic break were recorded, indicating proximity to the soma of the recorded neuron and excluding a fiber recording. This heterogeneity in waveforms questions the homogeneity of the neuronal population within the STN.

#### Expectation activity (responses at cue light)

##### The STN encodes the reward value according to the context (i.e., rewards available)

**Figure 4.**
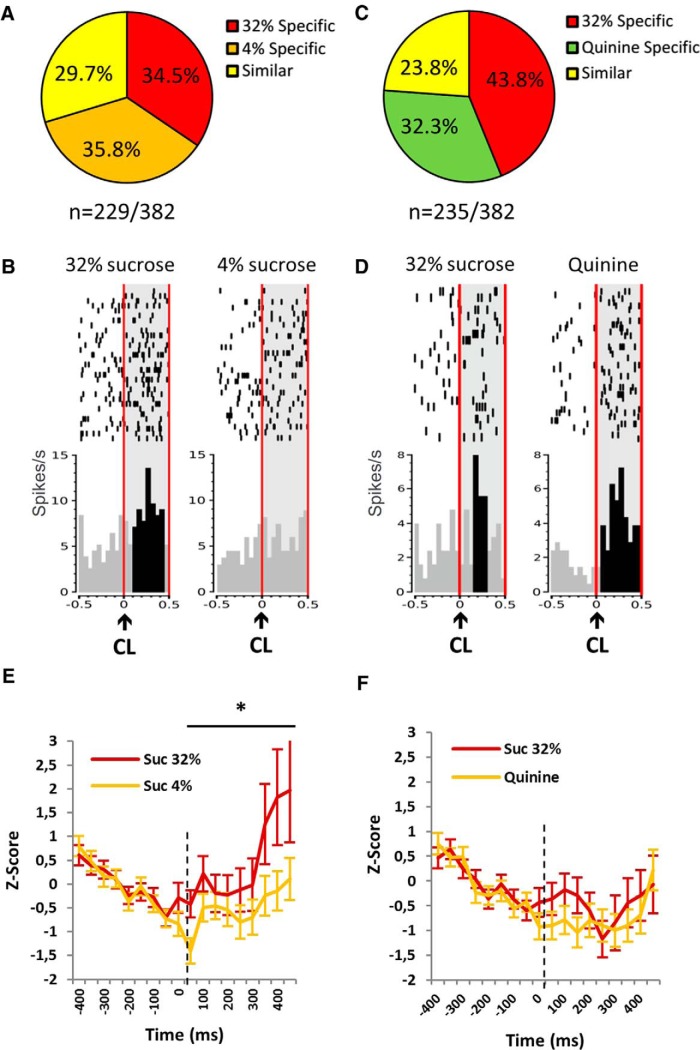
Responses of the STN neurons to the predictive cue lights (CLs). ***A***, ***B***, Proportions of the neuronal populations [32% sucrose specific (red), 4% sucrose specific (orange; ***A***), quinine specific (green; ***B***) and similar (yellow)] responding in the standard condition (***A***, *n* = 229 of 382) and during the quinine challenge (***B***, *n* = 235 of 382). ***C***, Example of the firing pattern of one STN neuron classified as 32% specific, showing increased activity to the CL predicting 32% sucrose (left) and no significant response to the CL predicting 4% sucrose (right). ***D***, Example of the firing pattern of another STN neuron classified as quinine specific showing increased activity to both the CL predicting 32% sucrose (left) and quinine (right), but with a higher increased activity to the CL predicting quinine. Rasters are centered on the occurrence of the CL (time = 0) that lasted 100 ms (two bins of 50 ms). The CL is indicated with a black arrow, and the light gray area delimited by the vertical red lines represents the period on which the bins were analyzed [0:500 ms]. The black bins represent the bins significantly different from the baseline ([−400:0 ms]). Top, Raster plot of spike firing on each trial (each row illustrates one trial), with the top row of dots corresponding to the first trial. Bottom, Mean firing rate across all trials, with a bin size of 50 ms. ***E***, ***F***, Average post-stimulus time histograms of the ﬁring rate (expressed as *z*-score) aligned with the cue light (0 ms) in standard condition (***E***) and quinine challenge (***F***) constructed with 50 ms bins for 32% (red) and 4% sucrose or quinine (orange). The lines represent the average PSTHs (mean ± SEM) of the whole population that respond to the CL. *Significant reward effect (*p* < 0.05). Suc, Sucrose.

In the standard condition, 60% of the recorded neurons (229 of 382) responded to the cue light (Fig. 4*A*). The majority of these neurons (70.3%; 161 of 229) were specific and were split into two equivalent subpopulations of “32% sucrose specific” (for an example, see Fig. 4*B*) and “4% sucrose specific” [34.5% (79 of 229) and 35.8% (82 of 229), respectively; χ^2^ = 0.09, *p* > 0.05 (NS)], whereas only 29.7% of the neurons (68 of 229) responding at this event were similar for both sucrose solutions (Fig. 4*A*). During the quinine challenge, 62% of the recorded neurons (235 of 382) responded to the cue light (Fig. 4*C*). Again, the majority of the neurons were specific (76.1%; i.e., 178 of 235), while only 23.8% of these neurons (56 of 235) were similar (Fig. 4*B*). However, unlike the standard condition, the population of 32% sucrose-specific neurons (43.8%; 103 of 235) was larger than that of “quinine-specific” neurons (32.3%; 76 of 235; χ^2^ = 6.58, *p* = 0.0103 (for an example, see Fig. 4*D*). This change in proportion to the various subpopulations of STN neurons suggests that the STN has integrated the aversive dimension of the quinine.

In standard conditions, activity (expressed as a *z*-score for the entire population) at the cue light predicting 32% sucrose was higher than that for 4% sucrose (ANOVA, reward effect: *F*_(1,321)_ = 3.858; *p* = 0.05; Fig. 4*E*). In contrast, after the replacement of the 4% sucrose by quinine, the activity at the cue light predicting 32% sucrose and quinine did not differ (ANOVA, reward effect: *F*_(1,312)_ = 0.681; *p* = 0.41; Fig. 4*F*). Interestingly, the activity at the cue light predicting 32% sucrose was significantly different from the standard condition (32% vs 4% sucrose) when quinine was on board (ANOVA, interaction effect: *F*_(1,322)_ = 1.889; *p* = 0.015). These results highlight the fact that responses to a same reward (32% sucrose) can differ depending on the context (defined regarding the two solutions on board) and that increased activity in the STN is associated with a favorable context.

**Figure 5.**
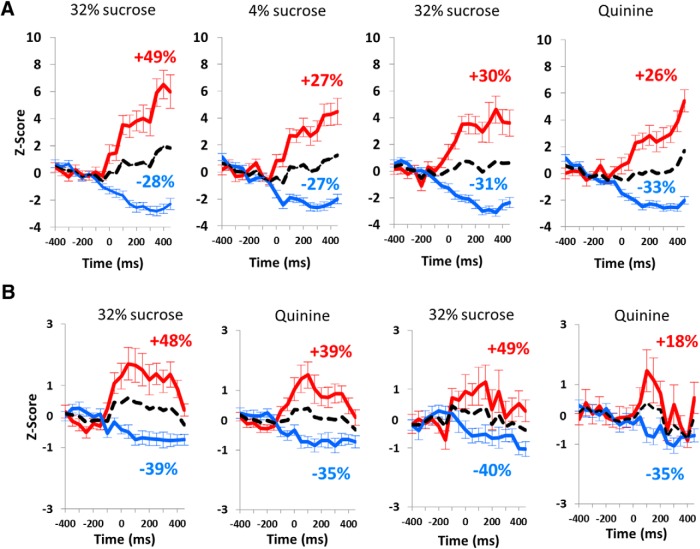
Excitation and inhibition at the cue light presentation and lever release. ***A***, Average *z*-scores (mean ± SEM) of the firing activity for STN neurons responding by an activation (red line) or an inhibition (blue line) to the cue light (time = 0 ms) in standard condition (left) and quinine challenge (right). ***B***, Average *z*-scores (mean ± SEM) of the firing activity for STN neurons responding by an activation (red line) or an inhibition (blue line) at the lever release (time = 0 ms) in correct trials (left) and incorrect trials (right). The black dotted lines represent the average activity of both activated and inhibited neuronal populations responding to the events. The percentages represent the mean variation of activity after each event for activated (red) and inhibited (blue) neuronal population. The *z*-scores are represented for the period on which the time bins were analyzed (−400:450 ms).

More specifically, when the response to the cue light predicting 32% sucrose was an excitation, there was a 49% increased amplitude of the excitation in the standard condition (Fig. 5*A*), while it reached only 30% increase during the quinine challenge (49% vs 30%; χ^2^ = 7.55, *p* = 0.006; Fig. 5*A*). Also interestingly, as for the mean activity, the excitation was higher for 32% sucrose than for 4% in the standard condition (49% vs 27%, respectively; χ^2^ = 10.27, *p* = 0.0014), but this difference was abolished during the quinine challenge (30% vs 26% increased *z*-score for quinine response; χ^2^ = 0.40, *p* = 0.53). For those neurons responding by an inhibition to the cue light predicting 32% sucrose, there was no significant difference in the amplitude of the response between standard and quinine challenge conditions (28% and 31% of decrease in the *z*-score, respectively; χ^2^ = 0.22, *p* = 0.64). The level of inhibition was equivalent between 32% and 4% sucrose (−28% vs −27%, respectively; χ^2^ = 0.11, *p* = 0.75) in the standard conditions and was slightly enhanced by quinine, although not significantly (−27% vs −33%; χ^2^ = 0.86, *p* = 0.36; Fig. 5*A*).

##### Evolution of selectivity from standard condition to quinine challenge: a “reset”

**Figure 6.**
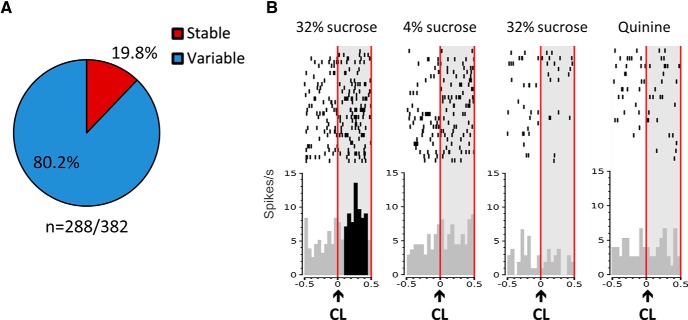
Evolution of the neuronal selectivity at the cue light (CL) according to the context (during challenge 1, when quinine replaces 4% sucrose). ***A***, Proportions of the 288 neurons responsive to the CL, showing a stable selectivity (red) or a change of selectivity (variable; blue) during challenge 1. Stable neurons (red) are neurons keeping the same selectivity after the quinine introduction (a 32% sucrose-specific neuron remaining 32% specific when quinine has replaced the 4% sucrose), while variable neurons (blue) are neurons changing their selectivity after the quinine introduction. ***B***, Example of the evolution of the firing pattern of an STN neuron classified as a 32% sucrose-specific neuron in the standard condition, showing increased activity in response to the cue light predicting the 32% sucrose (left) and no change of activity to the CL predicting 4% sucrose (right), which stopped to respond during the quinine challenge to both cue lights. Rasters are centered on the occurrence of the CL (time = 0) that lasted 100 ms (two bins of 50 ms). The CL is indicated with a black arrow, and the light gray area delimited by the vertical red lines represents the period on which the bins were analysed [0:500 ms]. The black bins represent the bins significantly different than the baseline (−400:0 ms). Top, Raster plot of spike firing on each trial (each row illustrates one trial), with the top row of dots corresponding to the first trial. Bottom, Mean firing rate across all trials; bin size is 50 ms.

Figure 6*B* illustrates a neuron responding only for the 32% sucrose during the standard condition and stopping to respond for the 32% sucrose during the quinine challenge. This illustrates the fact that the same cue could be encoded differently depending on the context (4% vs 32% sucrose and quinine vs 32% sucrose). To quantify the exemplified change in cue–reward encoding of STN neurons across context for each neuron, categorization in standard condition (similar, 4% sucrose specific, and 32% sucrose specific) was compared with that during the quinine challenge. For example, 5% of 32% sucrose-specific neurons (15 of 288) responding to the cue light in standard condition became quinine specific. Since sucrose 4% was replaced by quinine, it was expected that the 4% sucrose-specific neurons would be the main population changing specificity. In fact, there was a majority, almost 80% of neurons (231 of 288) changing their selectivity during the challenge (Fig. 6*A*). A further analysis has shown that this change of selectivity does not imply a systematic predictable change of selectivity, but rather a random reset for all neurons. Importantly, since in our example (Fig. 6*B*) the baselines seem different depending on the context, the comparisons among the baselines of all the neurons (*n* = 288) have been performed, and no difference has been found between standard condition and quinine challenge (ANOVA, condition effect: *F*_(1,616)_ = 0.128, *p* = 0.721).

#### Error-related activity

##### STN activity before the movement execution can predict the future premature release

**Figure 7.**
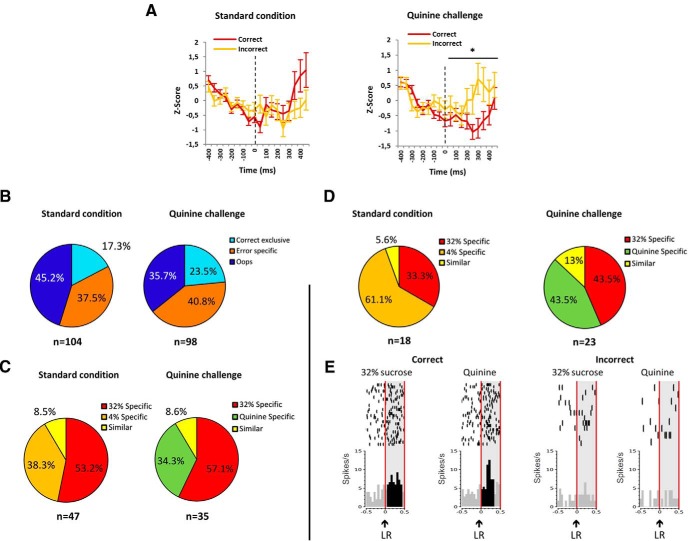
Proportions of neurons responsive to correct *vs* incorrect premature lever release. ***A***, Average post-stimulus time histograms (PSTHs) of the ﬁring rate (expressed as *z*-score) aligned with the cue light (0 ms, black dotted line), in standard condition (left) and quinine challenge (right) constructed with 50 ms bins preceding trials with future correct (red line) and incorrect (orange line) lever release. The lines represent the PSTHs (mean ± SEM) that respond to the cue light. *Significant reward effect (*p* < 0.05). ***B***, Proportions of neurons responding exclusively at lever release for correct trials (“correct exclusive neurons”; turquoise area), at lever release in both correct and incorrect trials but in a different manner (“error-specific neurons”; orange area), and responding exclusively at lever release for incorrect trials (oops neurons; dark blue area) in standard condition (left) and quinine challenge (right). ***C***, Selectivity to reward in oops neurons expressed as proportions in both standard condition (left) and quinine challenge (right) of 32% sucrose-specific neurons (red), 4% sucrose-specific neurons (orange), similar neurons (yellow), and quinine-specific neurons (green). ***D***, Selectivity to reward in exclusive correct neurons expressed as proportion in both standard condition (left) and quinine challenge (right) of 32% sucrose-specific neurons (red), 4% sucrose-specific neurons (orange), similar neurons (yellow), and quinine-specific neurons (green). ***E***, Example of the firing pattern of one STN neuron classified correct exclusive neuron showing increased activity at correct lever release only (left), in a similar manner for both 32% sucrose and quinine, but showing no response at lever release for incorrect trials (right), whatever the reward missed. Rasters are centered on the occurrence of the lever release (LR) (time = 0). The LR is indicated with a black arrow, and the light gray area delimited by the vertical red lines represents the period on which the bins were analyzed [0:500 ms]. The black bins represent the bins that were significantly different from the baseline [−400:0 ms]. Top, Raster plot of spike firing on each trial (each row illustrates one trial), with the top row of dots corresponding to the first trial. Bottom, Mean firing rate across all trials; bin size is 50 ms.

Neurons responding at the lever release in both correct and incorrect trials (premature lever release) were recorded. As previously described (Lardeux et al., 2009, 2013), among the 80.6% of the neurons (308 of 382) responding to the lever release, there was a higher baseline firing rate (i.e., over the 400 ms preceding the event itself) in correct than in incorrect trials (*t* test; mean ± SEM, 3.99 ± 0.27 and 3.48 ± 0.25 Hz, respectively; Student’s *t* test: *T*_(1,305)_ = −5.036; *p* < 0.0001; interaction correct (correct, incorrect trials) × lever release (baseline, postevent) effect: *F*_(1,305)_ = 9.966; *p* = 0.0017). This suggests that a lower basal firing rate can be predictive of a premature lever release. This is further confirmed by analysis of the *z*-score population response at the cue light, showing increased activity after the cue light for future incorrect trials during the quinine challenge (ANOVA success effect: *F*_(1,480)_ = 4.604; *p* = 0.032; Fig. 7*A*). This further confirms that increased activity following the cue light presentation is associated with a favorable situation (i.e., error during the quinine challenge, leading to avoidance of a possible quinine delivery).

##### Error-sensitive neurons: another subset of selectivity

Among the neurons responsive to lever release, 41.9% (160 of 382 neurons) responded differentially for correct and incorrect trials. As shown in Figure 7*B*, most of these neurons were exclusively responsive either to the incorrect (premature) lever release (i.e., oops neurons: 45.2% (47 of 104) and 35.7% (35 of 98) in standard and quinine conditions, respectively) or to both conditions, but differentially [37.5% (39 of 104) and 40.8% (40 of 98) in standard and quinine conditions, respectively), while other neurons responded only to the lever release in correct trials [i.e., “exclusive correct” neurons: 17.3% (18 of 104) and 23.5% (23 of 98) in standard and quinine conditions respectively].

The oops neurons, exhibiting a phasic response only at premature lever release (Lardeux et al., 2009, 2013) and the exclusive correct neurons, have been analyzed separately with regard to their reward selectivity.

##### Oops neurons: selectivity for the preferred reward

For both conditions (standard condition and quinine challenge), of all the recorded neurons responding at lever release, 22.1% (68 of 308) were oops neurons. In the standard condition, 47 oops neurons have been identified. The majority of these neurons (91.5%; 43 of 47) were specific, and mostly 32% sucrose specific (53.2% vs 38.3%; χ^2^ = 4.47, *p* = 0.0345; Fig. 7*C*). This difference was further enhanced when a 4% sucrose solution was replaced by quinine and 36.5% of oops neurons were found (35 of 96 neurons; 57.1% specific 32% vs 34.3% “specific quinine”; χ^2^ = 10.53, *p* = 0.0012; Fig. 7*C*). This suggests that the oops neurons specially encode a response related to the loss of the preferred reward.

##### Exclusive correct neurons encode rewards depending on the context

In contrast to the oops neurons, when neurons respond exclusively at correct lever release, their selectivity for rewards depends on the context. For both conditions (standard condition and quinine challenge), of all the recorded neurons responding at lever release, 11.7% (36 of 308) were exclusive correct neurons. In the standard condition, 18 of these neurons have been identified. The majority of these neurons (94.4%; 17 of 18) were specific with a majority for the 4% sucrose specific compared to the 32% sucrose specific (61.1% vs 33.3%, respectively; χ^2^ = 15.48, *p* < 0.0001; Fig. 7*D*). This distribution changed when a 4% sucrose solution was replaced by quinine and 23 exclusive correct neurons were found. Thus, the majority of these neurons (87%; 20 of 23) were specific and equally distributed between 32% sucrose specific and “quinine specific” (43.5% specific 32% sucrose vs 43.5% specific quinine; χ^2^ = 0, *p* = 1; Fig. 7*D*; for an example of an exclusive correct neuron with similar response to 32% sucrose and quinine, see Fig. 7*E*).

##### Activation or inhibition at lever release for correct and incorrect trials

During the quinine challenge, the neurons responding to the lever release in 32% sucrose trials did not show differential activity between correct and incorrect trials (48% and 49%, respectively, for excitations; −39% and −40%, respectively, for inhibitions; Fig. 5*B*). Similar to the responses at the cue light, the excitation following correct lever release for 32% sucrose tended to be higher than that for quinine solution (48% vs 39%, respectively), and was significantly higher in incorrect trials (49% vs 18%; χ^2^ = 18.61, *p* < 0.0001). Interestingly, while there was no difference in the level of inhibition between correct and incorrect trials for quinine (−35% for both types of trials), the level of excitation was significantly reduced for the incorrect trials (18% increased activity) when compared with the correct trials for which quinine was expected (39% increased activity; 39% vs 18%; χ^2^ = 8.68, *p* = 0.0032).

##### STN encodes reward omission

**Figure 8.**
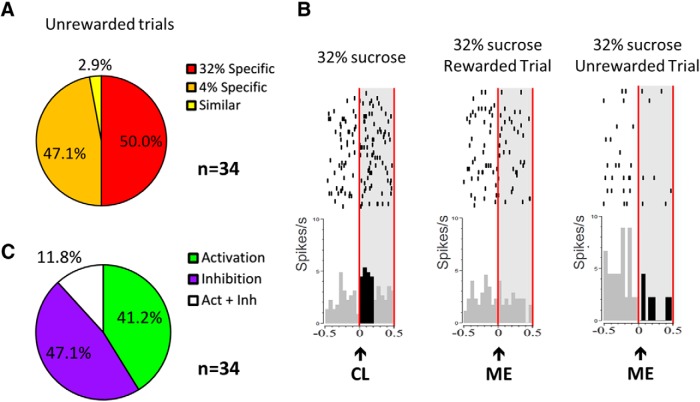
Responses of the STN neurons at the magazine entry during challenge 2, when the rewards were omitted in 20% of cases. ***A***, Proportions of the different neuronal categories responding at magazine entry for the unrewarded trials [32% sucrose specific (red), 4% sucrose-specific (orange), and “similar” (yellow)]. ***B***, Example of the firing pattern of one STN neuron classified as 32% sucrose specific at the cue light (CL; left), and its response to 32% sucrose delivery at the magazine entry (ME; middle) and at the ME when 32% sucrose was omitted (right). Rasters are centered on the occurrence of the CL (time = 0) that lasted 100 ms (2 bins of 50 ms; left) and the ME (time = 0; middle and right). The CL and the ME are indicated with a black arrow, and the light gray area delimited by the vertical red lines represents the period on which the bins were analyzed (0:500 ms). The black bins represent the bins significantly different of the baseline [−400:0 ms]. Top, Raster plot of spike firing on each trial (each row illustrates one trial), with the top row of dots corresponding to the first trial. Bottom, Mean firing rate across all trials; bin size is 50 ms. ***C***, Proportions of activated (bright green area), inhibited (violet area), and mixed (activation and inhibition; white area) neurons responding to magazine entry for the unrewarded trials.

The surprise effect of not finding an expected reward is likely to occur at the magazine entry during omission trials. STN activity was thus compared for rewarded trials versus unrewarded trials for both sucrose solutions (4% vs 32% sucrose) at magazine entry. Among the neurons responding at magazine entry (*n* = 366), 54.1% (198 of 366) responded differentially for rewarded and unrewarded trials. Some of these neurons responded only for unrewarded trials (*n* = 34). Nearly all of them (97.1%; 33 of 34) were specific to one omitted reward, but in equivalent proportion for 32% and 4% sucrose [50% vs 47.1%; χ^2^ = 0.06; *p* > 0.05 (NS); Fig. 8*A*]. Although phasic inhibition could be observed like in DA neurons (Fig. 8*B*, right), in general, reward omission induced inhibition and activation in equivalent proportions (47.1% vs 41.2%, respectively; χ^2^ = 0.24, *p* > 0.05; Fig. 8*C*). Further analysis showed that STN neurons did not show a differential response graded by the size of the prediction error (ANOVA, undelivered reward size effect: *F*_(1,79)_ = 0.017, *p* = 0.896), suggesting that, if STN neurons encode omission, they do not encode proper reward prediction error like dopaminergic neurons.

## Discussion

### Positive and negative reinforcers can modulate STN activity via various subpopulations

The present study shows that STN neurons can encode the expectation of an aversive reinforcer (quinine), as well as a positive reinforcer (sucrose). The neuronal modulation observed in the STN in the unfavorable context using quinine is in line with its role in affective responses, which have recently been demonstrated (Pelloux et al 2014). Since it has been shown that both orbitofrontal cortex (OFC) and DA neurons modulate their activity in response to aversive reinforcers and their predicting cues (Thorpe et al., 1983; O’Doherty et al., 2001; Roesch and Olson, 2004; Schoenbaum and Roesch, 2005; Hosokawa et al., 2007; Brischoux et al., 2009; Matsumoto and Hikosaka, 2009), the aversive coding properties of the STN could come from DA neurons and/or OFC neurons through the hyperdirect pathway (Nambu et al., 2002; Haynes and Haber, 2013). The behavioral data, RT and MT, were modulated by the reinforcers in the various conditions tested, suggesting that rats have well associated each light with each reward. They also reveal that the context (favorable, 4% vs 32% sucrose; unfavorable, quinine vs 32% sucrose) affects general speed and accuracy for both rewards, suggesting that both the valence of the reinforcers and the context (which will be discussed further below) influenced the performances of the rats. In line with the studies of Goodwin and Amit (2000) or Pelloux et al. (2006), who reported that rats avoided quinine solutions at lower concentrations (ranging from 9 × 10^−9^ to 4 × 10^−5^ m) than that used here (0.02 m), we confirmed strong aversive reactions to this concentration of quinine with the taste reactivity measures. Thus, quinine can be considered as aversive in the electrophysiological experiment, and the contrast between the rewards should be considered larger when quinine replaced the 4% sucrose solution in the challenge 1.

As previously shown (Lardeux et al., 2009, 2013), STN neurons are mostly specific to one given reward in equivalent proportion for each of the two rewards available. This was based on the responses observed at the cue light presentation. Since STN is well known for its involvement in motor behavior, one might argue that responses here at the cue light might not be strictly reward-related responses. Nevertheless, the responses to the cue light were recorded during lever holding, so the motor behavior was held constant and could allow dissociation between reward-related activity and motor-related activity. Moreover, if motor preparation cannot be excluded before lever release, because cue light information leads to the speeding of movement shown on the various behavioral measures, it should nevertheless be limited since the required movements (i.e., release of the lever and reaching the magazine) remain the same whatever the expected reward. Any change after the cue light might thus be associated partly with motor preparation, but modulated by a motivational change predicted by cue light and associated with a specific reinforcer.

The populations of neurons specific to the cue associated with either 32% or 4% sucrose solutions were in equivalent proportions in the standard condition. Of interest, when replacing the 4% sucrose solution by quinine, and therefore increasing the contrast between rewards, the proportion of selective 32% neurons became greater than that of “selective quinine.” In this high-contrast situation, it is likely that a 32% sucrose-associated cue may gain higher incentive value than in a lower contrast situation. Consequently, the number of mobilized STN neurons may in fact encode the relative value of the predictive cue (utility) more than the absolute value of the reinforcer, as further discussed below.

### Sensitivity to the context

To better understand the type of reward-related information encoded by the STN, we have used different contexts, as follows: the standard condition (4% vs 32% sucrose, a favorable context using only positive reinforcers) and the quinine challenge (quinine vs 32% sucrose, an unfavorable context).

As mentioned above, the general speed, as measured by movement time, was affected during the quinine challenge (i.e., the unfavorable context), even for the trials rewarded by a 32% sucrose solution. It may thus be argued that repetitive presentation of quinine to the animals could have led to devaluation of the positive valence of the sucrose solution or to an increase of the valence of the quinine. In favor of this point, we have observed that the general STN neuronal activity recorded at the cue light predicting 32% sucrose was reduced during the quinine challenge when compared with that in the standard condition. This effect was in fact mostly due to a reduction of the excitatory responses. The same reward placed in an unfavorable context can thus induce a different effect on the STN neuronal activity.

In the classic devaluation protocol using lithium chloride injection, the injection is directly paired with the positive reinforcer. In contrast, in our experiment, animals could not consume quinine and sucrose solutions in the same cup; they had, therefore, a distinct representation of each reward associated with each cup. Furthermore, since animals always consumed the sucrose when available, there was no possibility of accessing sucrose during quinine trials. The possibility that quinine could have also left a lingering aftertaste in the mouth, thus leading to devaluation of sucrose, is limited since, after a few trials, the animals did not consume the quinine solution. The possibility of devaluation for sucrose solution was thus minimized. Our results may show the opposite phenomena. Indeed, the fact that rats made fewer errors when quinine and 32% sucrose were on board might suggest higher motivation or higher sensitivity to contrast between the two rewards during the quinine challenge and, therefore, increased attention to perform correctly. Alternatively, it could also be due to the fact that standard conditions were always applied before the challenge at the beginning of the session, when the rats are excited and therefore more prone to premature lever release. By the time they reached the challenge (15 min or 60 trials), they were more willing to wait and less prone to make errors.

The specificity of the responses at the cue light for one reward seems then to depend on the context (i.e., the rewards on board) and is not set permanently. Here, only a few neurons maintained their specificity to the same reward when 4% sucrose was replaced by quinine, even those neurons specific to the reward that remained unchanged (32% sucrose.). The random resetting of specialization suggests that the neuronal response to an anticipated and/or given reward in the STN depends of the alternative reward available, and that the STN reassesses the relative value of each reward depending on the value of the alternative reward or context.

These results are supported by previous studies (Lardeux et al., 2009, 2013) showing differences between populations of STN neurons after different challenges varying the relative valence of different rewards. Previous studies performed in the OFC suggest that OFC neurons encode the relative preference of rewards (Tremblay and Schultz, 1999; Hosokawa et al., 2007) and also positive and negative outcomes (Schoenbaum et al., 1998), and these properties seem to be shared by the STN. Similarly, PFC has been proposed to encode “contextual information concerning which kind of reward may be delivered in the following trial” (Watanabe et al., 2002). Indeed, lateral PFC neurons encode the contextual information between different conditions: go/no-go or rewarded versus unrewarded trials (Watanabe and Sakagami, 2007; Schoenbaum and Eichenbaum, 1995a,b Feierstein et al., 2006). It is therefore possible that the modulations observed here at the level of the STN are mediated via these cortical territories sending direct projections to the STN via the so-called hyperdirect pathway.

As described and illustrated in Figure 6, at the moment the 4% sucrose solution is replaced by quinine, there is a reset of the specialization of STN neurons. This reset has not been described in our former studies when cocaine was replaced by saline or sucrose was replaced by water, although the rewarding properties of the same cue were also diminished. The reset observed in the present study could thus reflect the aversive versus appetitive change that operates when changing from a favorable to an unfavorable context.

The fact that the level of activation after the cue light was the highest for the 32% sucrose in the standard condition suggests that in a favorable context (4% vs 32% sucrose) the preferred reward induces the strongest excitation. An excitatory response following the cue light in the STN might thus be associated with a favorable situation. In line with this hypothesis, the STN activity was increased after the cue light for future incorrect trials in the quinine challenge, as the favorable situation would be to avoid a possible delivery of aversive solution.

### Encoding of execution error and reward prediction error

It was shown here that a lower basal firing rate preceding lever release was predictive of future error (i.e., premature lever release). Inactivation of the STN has been previously shown to result in premature responding (Baunez et al., 1995; Baunez and Robbins, 1997, 1999). Altogether, these results strongly support a causal involvement of reduced STN activity in premature responding. At lever release, the neuronal response recorded could reflect various events such as execution monitoring, sensory integration, motor preparation, or reward anticipation. It is nevertheless unlikely that these responses were related to motor preparation. Since the movement to reach the magazine is similar whatever the reward, neural responses should be independent of the reward. This is not the case, as when the lever release is correct, >90% of the neurons responding then are reward selective. It appears thus that the anticipation of the reward is taken into account at the lever release.

The fact that the activity at lever release changes for correct and incorrect responses thus suggests that STN may process error monitoring under the influence of the various reinforcers. Indeed, oops neurons were shown to specially respond to errors made before the preferred reward. By responding phasically at the premature level release made before the preferred reward, the oops neurons could encode the frustration of missing the preferred reward. In contrast, the exclusive correct neurons, by encoding both rewards, depending on what is available, could encode the expectation of either the negative quinine or the positive 32% sucrose in a defined context.

The ability of the STN to respond differentially to correct and incorrect trials, and also differentially for the preferred reinforcer, highlights its role in the encoding of the relative preference for reward. Some studies have shown the existence of neurons responding specifically during behavioral errors in the PFC, the globus pallidus, and the nucleus accumbens (Watanabe, 1989; Arkadir et al., 2004; Amiez et al., 2005; Taha et al., 2007). Interestingly, the anterior cingulate cortex in particular encodes the error depending on reward prediction size (Amiez et al., 2005), and may thus, along with the midbrain DA neurons (Schultz and Dickinson, 2000), drive the neuronal responses observed in the STN during error trials via the hyperdirect pathway linking the PFC to the STN. Specific studies will be necessary to validate or not validate this hypothesis.

Finally, the present study has also shown that STN neurons respond to reward omission. Indeed, some STN neurons show specific activation or inhibition at magazine entry in response to the omission of an expected reward, while they were unresponsive when the reward was actually delivered. Interestingly, the opposite situation, such as neurons responding only in the rewarded trials and not in the unrewarded trials, could be observed. Therefore, not only can STN neurons adapt to unexpected reward in the case of changes [4% sucrose replaced by quinine or sucrose replaced by water (Lardeux et al., 2009) or cocaine replaced by saline (Lardeux et al., 2013)], but they also can be reactive to unexpected reward omission. Together, these properties are in favor of a role for STN in RPE processes. RPE is a classic characteristic of DA neurons, in which they show excitation at unexpected reward delivery and inhibition at omission of reward (Schultz, 1998; 2002). Since the STN receives DA inputs, it is possible that this RPE in STN could be under the control of the DA inputs, but this remains to be investigated. The fact that the modulation within STN is not as clear as it is for DA neurons (STN neurons can also be activated by unrewarded trials) suggests that there may be another influence than simply DA in the RPE STN responses.

In conclusion, we report here that STN neurons encode relative reward values depending of the context [favorable or not (using an aversive reinforcer)] and adapt to changes in the rewards available, as well as reward omission, suggesting a role in reward prediction error. These results show that STN shares a lot of cortical properties, as supported by other studies (Baunez and Lardeux, 2011; Chudasama et al., 2003; Haynes and Haber, 2013). It thus seems that STN evaluates the relative preference of a reinforcer and integrates error signal to possibly help select the most appropriate response for the favored reward possible in a favorable context. These abilities of STN neurons to encode different types of information to modulate their activity position the STN at a critical position for decision-making processes. It is therefore not surprising to see, not only the list of possible applications of STN DBS growing over time, but also the list of possible complications associated with STN manipulations. These latter are particularly important to consider in the current development of DBS surgery in conditions ranging from neurological to psychiatric disorders (Krack et al., 2010).

## Synthesis

The decision was a result of the Reviewing Editor Philippe Tobler and the peer reviewers coming together and discussing their recommendations until a consensus was reached. A fact-based synthesis statement explaining their decision and outlining what is needed to prepare a revision is listed below. The following reviewer(s) agreed to reveal their identity: Kuei Tseng

The reviewers would like you to include the additional z-score figure (sent in the rebuttal) in the paper.

Moreover, several points have been raised which you should address.

Describe the main contribution(s) of the paper more clearly in all parts of the paper, including results and discussion - at the moment a narrative arc is missing that connects all parts of the paper. In the introduction it is now implied that the main contribution is the characterization of CS-responses. However, it is rightly mentioned that these responses have been reported before. By contrast, in the discussion it is implied that the main contribution is the reset. However, based on the results the implication is that the reset is not well understood. So it is as if the introduction, results and discussion have been written by different people who did not know what the others are writing.

Related to major point 1, the limitations of the study should be acknowledged more clearly:

It is unclear whether STN neurons encode salience (in the sense of absolute value) or whether some are activated by positive value whereas others are activated by negative value. For example, it could simply be that 4% sucrose is close to neutral and that there are neurons that code value or salience positively or negatively. These neurons would show a response for one condition and not for the other. The problem here is with the design in that only two concentrations/solutions were tested at any one time and contexts did not change back and forth.

Around line 407 a statistical comparison between 32% sucrose before versus after quinine challenge should be performed. Even if that is significant, they probably cannot exclude the possibility that the sucrose simply has less value as soon as quinine is introduced, which could at least partly explain the change in responding, even though there appeared to be a random reset. Although this possibility is now discussed around line 580, it is dismissed. Yet, it seems conceivable that quinine leaves a lingering aftertaste in the mouth that could interact with sucrose. Reversal learning procedures could have been employed to investigate this. Another possibility is that the reference point changed as soon as quinine was introduced, which together with differential neuronal sensitivity to such a change may explain some of the effects. The main problem here is that the study fails to clarify what is going on at the reset.

The number of gapes for quinine should be statistically compared against zero in the taste reactivity test.

The changes around line 540 do not seem to make sense in light of the previous statement that *differences in motor behavior* indicate that the animals have learned the meaning of cue lights. It seems that one would have to acknowledge the possibility that differences in motor preparation may exist (which would be fine), unless this possibility is addressed with finer-grained analysis.

More minor:

Line 11: Typo for "Specialization"

Line 55 to 59 need to be rewritten. One suggestion is the following:

In cue directed instrumental conditioning or associative learning, the association of a positive or negative US (sucrose or quinine) with a CS attaches positive or negative value to that CS (Konorski, 1948; Skinner, 1966a, b).

Please replace "oops" with a more scientific term (by the way, it appears that these neurons could also encode the turning off of the house light?)

Delete "positive" in "positive rewards". Moreover, in lines 342, 343, 353, 409, etc. "reward" should be replaced with "solution". Reward is positive by definition and they argue that quinine is negative, so it should not be implied that quinine is a reward.

Lines 345/346: The fact that overall movement times slow down as such is not an indication that the animals have properly learned the association between different lights and reinforcers, all it shows is that they have realized that the overall value of the situation has decreased.

"stucked" is not English.
